# Biology, Society, or Choice: How Do Non-Experts Interpret Explanations of Behaviour?

**DOI:** 10.1162/opmi_a_00098

**Published:** 2023-08-20

**Authors:** Daniel Nettle, Willem E. Frankenhuis, Karthik Panchanathan

**Affiliations:** Institut Jean Nicod, Département d’études cognitives, Ecole Normale Supérieure, Université PSL, EHESS, CNRS, Paris, France; Population Health Sciences Institute, Newcastle University, Newcastle, UK; Department of Psychology, Utrecht University, Utrecht, The Netherlands; Max Planck Institute for the Study of Crime, Security and Law, Freiburg, Germany; Department of Anthropology, University of Missouri, Columbia, MO, USA

**Keywords:** core cognition, intuitive psychology, intuitive biology, explanation, science communication

## Abstract

Explanations for human behaviour can be framed in many different ways, from the social-structural context to the individual motivation down to the neurobiological implementation. We know comparatively little about how people interpret these explanatory framings, and what they infer when one kind of explanation rather than another is made salient. In four experiments, UK general-population volunteers read vignettes describing the same behaviour, but providing explanations framed in different ways. In Study 1, we found that participants grouped explanations into ‘biological’, ‘psychological’ and ‘sociocultural’ clusters. Explanations with different framings were often seen as incompatible with one another, especially when one belonged to the ‘biological’ cluster and the other did not. In Study 2, we found that exposure to a particular explanatory framing triggered inferences beyond the information given. Specifically, psychological explanations led participants to assume the behaviour was malleable, and biological framings led them to assume it was not. In Studies 3A and 3B, we found that the choice of explanatory framing can affect people’s assumptions about effective interventions. For example, presenting a biological explanation increased people’s conviction that interventions like drugs would be effective, and decreased their conviction that psychological or socio-political interventions would be effective. These results illuminate the intuitive psychology of explanations, and also potential pitfalls in scientific communication. Framing an explanation in a particular way will often generate inferences in the audience—about what other factors are not causally important, how easy it is to change the behaviour, and what kinds of remedies are worth considering—that the communicator may not have anticipated and might not intend.

## INTRODUCTION

In the social and behavioural sciences, and indeed in everyday talk, we explain human behaviour in a number of different ways. We might for example say that the actor wanted to do it; that they chose to do it; that they did it because of what it meant to them; that they had a predisposition to doing it; that they were constrained to do it by social factors or cultural expectations; that the behaviour was driven by hormonal or physiological mechanisms; or that it promoted the person’s survival or reproduction. The topic of the present study is the way non-experts interpret these different ways of explaining. Which ones are perceived as meaning the same thing? Are they perceived as compatible or not? And what does the audience infer about the behaviour from the fact that the speaker used one way of talking rather than a different one?

We will refer to the different ways of talking about behaviour as *explanatory framings*, related to the notion of frame or framing employed in communication studies (Entman, 1993). An explanatory framing is defined by the kind of process or entity offered as the explaining factor. We use the term explanatory framing rather than explanation because explanations are tokens, whereas explanatory framings are types. For example, ‘Paul smokes because of a lack of inhibitory activation in the dorsolateral prefrontal cortex’ and ‘Paul smokes because of a strong reward signal in the amygdala in response to tobacco cues’ are different explanations, but the same explanatory framing (the explaining factor is a process in the brain).

The explanatory framings (henceforth, for brevity, framings) that we study here are those we encounter commonly in reading the literature in the social and behavioural sciences, and popular discussions of that literature. Our set of framings cross-cuts typologies of explanations that have been offered by philosophers. In some cases, the framings differ in *level* of explanation (Marr, 1982). For example, an explanation with a brain-based framing might represent the same facts as one with a cognitive framing, but at a lower or more reductive level (as discussed by Hopkins et al., 2016). In other cases, the framings differ in *stance* (in the sense discussed by Dennett, 1987). For example, desire or choice framings take the intentional stance; brain or hormonal-based framings take the physical stance; and adaptive evolutionary explanations take the functional stance. But in some cases, two framings share the same level and stance but we nonetheless characterize them as different: for example, a hormonal and a brain-based explanation; or an explanation in terms of choice and one in terms of desire. People might interpret such pairs as near-synonyms, but this is something we wish to discover rather than assume.

Explanations for the same phenomenon with different framings are often, in fact, mutually compatible, in the sense that one being true does not necessarily make the other false. The causal chains leading to specific behaviours have many links. For example, social-structural factors can exert properly causal influence on behaviour, but they generally do so by constraining the choices and consequences for individual actors. The individual actors still have to make choices within the constraints, so their agency is also causal (Haslanger, 2016; Ross, 2023). And the choices and agency of an individual actor can be unpacked at lower mechanistic levels, such as that of neural implementation (Marr, 1982). That implementation may be structured the way it is because of past consequences for survival and reproduction (Tooby & Cosmides, 1989). The veracity of such evolutionary explanations in terms of adaptive function does not make explanations for the same phenomenon in terms of mechanism false (Tinbergen, 1963). Indeed, much of the work in behavioural biology resides in integrating the two. Thus, while not every pair of explanations is mutually compatible, having very different framings does not necessarily rule compatibility out.

However, the fact that explanations with different framings can be compatible does not mean that they are cognitively equivalent. Explanation-giving is a communicative activity (Hilton, 1990; Kirfel et al., 2022), and follows the pragmatic and cooperative principles common to human communication in general (Sperber & Wilson, 1995). That is, if a speaker offers an explanation with framing X, where other framings would be possible, the audience may infer that the speaker believes that framing X is not just a true explanation, but the most relevant one. Explaining is closely tied to offering causes, and the causal explanations that people prefer are generally those that identify difference-making factors (Hitchcock & Knobe, 2009; Lombrozo, 2010; Quillien, 2020). That is, an explanation for X is a useful one if it makes salient the factor that makes the difference between X happening and not happening. Put another way, good explanations economically characterize a possible intervention on the world that would change the outcome (Hitchcock & Knobe, 2009; Woodward, 2003). If a speaker, asked why a recent car crash happened, gives a detailed physical account of the kinetics of the cars, hearers will assume that they do not know that one of the drivers was asleep, or they would have mentioned that first. Thus, the choice of one framing over another logically compatible one may make a considerable difference to what hearers infer about where the key difference-makers lie, and hence whether or how it would be possible to intervene.

Previous research on framings has tended to assume higher-order groupings: for example ‘biogenetic’ versus ‘psychological’ (Haslam & Kvaale, 2015); ‘individualistic’ versus ‘societal’ (Cozzarelli et al., 2001); ‘dispositional’ versus ‘situational’ (Piff et al., 2020); or ‘functional’ versus ‘mechanistic’ (Lombrozo & Gwynne, 2014). Because each of these studies focuses on one of these dichotomies, chosen a priori, each study uses only a subset of the framings routinely encountered in the social and behavioural sciences. Moreover, they assume that the higher-order groupings are psychologically real for their participants. The innovation of our studies is to use a larger set of framings, and to allow the higher-order grouping to emerge from the participants’ responses, rather than assuming it.

In what follows, Study 1 examines which framings are judged as more and less similar to one another, and more and less compatible. Study 2 examines the inferences people make beyond the information given when explanations with different framings are used, particularly about the malleability of the behaviour and the locus of causal responsibility. Studies 3A and 3B examine inferences about interventions: what differences does it make to people’s views about how a behaviour could change, when an explanation is framed one way rather than another?

## STUDY 1

Study 1 explored perceived similarity and compatibility of explanations for a behaviour. We sought to determine which kinds of explanations were perceived as meaning roughly the same thing, and which ones were different. Likewise, we wished to know to what extent having an explanation with one framing ruled out the truth of an explanation with a different framing.

As for compatibility, we can define two possible extreme scenarios. At one extreme, people could be total ‘compatibilists’, believing that the truth of an explanation with one framing never rules out the truth of explanations with any different framing. At the other extreme, they could be hard ‘incompatibilists’, believing that the acceptance of the truth of one framing necessarily means rejecting the truth of explanations with any other framing. Previous research suggests an intermediate situation: different types of explanation for the same phenomenon do often coexist in people’s minds (Blanchard et al., 2022; Legare & Visala, 2011; Shtulman & Lombrozo, 2016), but ‘explanation discounting’, whereby the availability of one explanation makes other explanations seem less plausible, has also been documented (Kelley, 1973; Sloman, 1994). Explanation discounting can also happen at the level of framings (Heussen, 2010). For example, among both lay people and physicians, the more someone believes a disorder has biological causes, the less they believe it has psychological causes, and vice versa (Ahn et al., 2009; Marsh & Romano, 2016). Thus, it is likely that certain combinations of framings will discount one another more strongly than other combinations do.

UK general-population adults read vignettes describing a behaviour and were then asked to consider different explanations. They rated either how similar those explanations were to one another (half the participants), or how compatible they thought they were (the other half). This design allowed us to map the landscapes of perceived similarity and compatibility, and investigate how similarity and compatibility related to one another. We also used cluster analysis to determine how many types of framing there were.

The study was exploratory rather than confirmatory. However, we were guided by one possible hypothesis: explanations would be seen as similar, and compatible, to the extent to which they activate the same core cognitive system. Some psychologists argue that humans are endowed with a small number of separate core cognitive systems for dealing with different classes of entity (Spelke & Kinzler, 2007). From early childhood onwards, these give rise to several distinct systems of intuitive knowledge and spontaneous inference: intuitive physics, which deals with objects and their motion (Spelke, 1990); intuitive biology, which deals with species of plants and animals (Atran, 1998); intuitive psychology, which deals with the beliefs, desires and goals of human agents (Kamps et al., 2017); and, perhaps, intuitive sociology, which deals with social groups, group membership and social norms (Shutts & Kalish, 2021). Each of these systems produces a characteristic, and different, type of intuitive reasoning. In principle, human behaviour could activate any or all of these systems: humans are, after all, physical objects, animals, individual agents, and members of social groups. Which system(s) are active might depend on the way the behaviour is framed. If this hypothesis is correct, we would expect explanations to cluster for similarity and compatibility according to which feature of humans—their animal-like embodiment, their psychological states, or their membership of social structures or networks—was made most salient.

### Methods

#### Ethics.

All studies received ethical approval from Newcastle University Research Ethics Committee (approval 16509/2021).

#### Pre-registration.

Although this study was exploratory, we pre-registered methods, materials, and planned analyses at: https://osf.io/8ba5m.

#### Participants.

Participants (200 UK resident adults with first language English) were recruited via online platform Prolific (www.prolific.co). Mean age was 39.9 years (*SD* 13.0), with 104 women and 96 men. Participants were mostly non-students (145 non-students, 22 students, 33 not stated), in employment (124 employed full or part-time, 32 not currently employed, 34 not stated). Participants received £2.50 for taking part. Six participants were excluded for failure to fully complete the survey, leaving 194.

#### Design.

**Figure 1.**
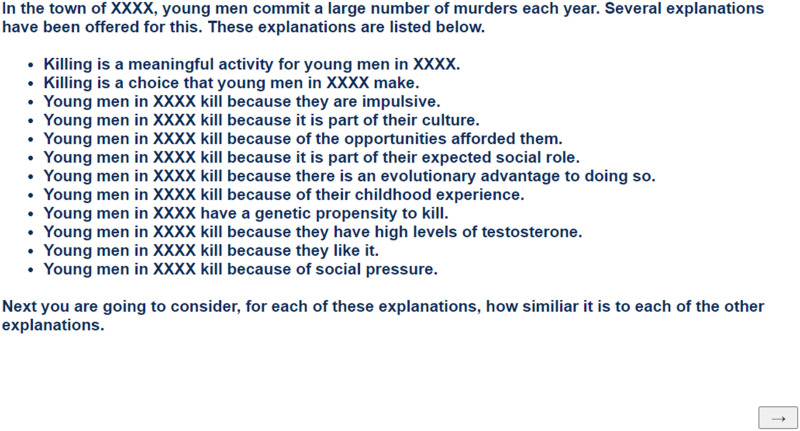
Screen capture from the study, showing one of the brief vignettes and its 12 explanations.

Participants were allocated, in a between-subjects design, to one of four different brief vignettes, and one of two different response conditions (similarity or compatibility). Each vignette described a behavioural phenomenon that was common in a particular population, respectively: homicide, teenage parenthood, land diving (a risky display behaviour), and blood blessing (a precautionary behaviour for dangers). At the end of the vignette, participants saw 12 ‘explanations that have been offered for the behaviour’. Figure 1 shows the vignette and explanations for one of the four cases. The wording for the other three vignettes is given in the pre-registered protocol (https://osf.io/8ba5m). The explanations were framed in terms of: meaning, choice, a psychological trait, culture, social structure, social roles, evolutionary advantage, physiology, childhood experience, genetic propensity, motivation, and social pressure. From here on, we refer to the psychological-trait explanation as ‘trait’, because ‘psychological’ will be later used as a superordinate category. The precise wording of each explanation was slightly adapted to the specific vignette.

Following the initial presentation of the vignettes and explanations, one explanation (the focal explanation) was placed at the top of the screen. The participant was then asked to rate, for each of the other 11 explanations, either ‘how similar you think it is to the explanation listed at the top of the page’ (similarity condition; 0–100 slider anchored with ‘totally different’ and ‘totally similar’), or ‘how compatible you think it is with the explanation listed at the top of the page’ (compatibility condition; 0–100 slider anchored with ‘totally incompatible’ and ‘totally compatible’). In the compatibility condition, the initial instructions further specified that ‘by compatible, we mean that both explanations could be true at the same time’.

The rating procedure was then repeated a further 11 times with each of the explanations in turn serving as the focal explanation. Thus, each participant completed a total of 132 ratings (11 other explanations × 12 focals). Every possible pairing of explanations appeared twice; once with the first explanation of the pair as the focal, and once with the second explanation as the focal. As a robustness check prior to the main analysis, we examined the correlation between the rated similarity or compatibility the first time the participant rated the explanation pair, and the second time. In the pre-registration, we specified that we would exclude any participant for whom the correlation between their first set of ratings and their second was less than 0.7. Observed correlations for each participant were lower than anticipated (similarity condition: mean *r* = 0.39; compatibility condition, mean *r* = 0.43). However, the correlations between the across-participants *average* rating the first time and the second time were extremely high (similarity condition: *r* = 0.93; compatibility: *r* = 0.79). We therefore adjusted our pre-registered exclusion criterion and excluded any participant who lacked a significant positive correlation between their first set of ratings and their second. This excluded 58 participants, leaving 136. In the post-exclusion dataset, the correlation between across-participants average ratings the first and second time were *r* = 0.96 (similarity condition) and *r* = 0.89 (compatibility condition). All subsequent analyses use ratings averaged across participants and across the first and second time of rating.

#### Data Analysis.

Data and code for all studies are available at: https://osf.io/wte2c/. Similarity ratings for pairs of framings were well correlated across vignettes (inter-vignette correlations 0.68–0.88), as were compatibility ratings (inter-vignette correlations 0.67–0.84). All subsequent analyses were performed on the data pooled across vignettes. First, we used multidimensional scaling to create a map of rated similarity, using R package ‘smacof’ (see Jones et al., 2018). We then used the configuration matrix from the multidimensional scaling output to perform *k*-means clustering analysis to detect clusters in the similarity space. We determined the optimal number of clusters to extract using the function fviz_nbclust() from the ‘factoextra’ R package (Kassambara & Mundt, 2020). We then repeated the multidimensional scaling and clustering for rated compatibility. Finally, we examined how rated compatibility related to rated similarity. We examined how rated compatibility differed by the respective similarity cluster membership of the two framings in question.

### Results

**Figure 2.**
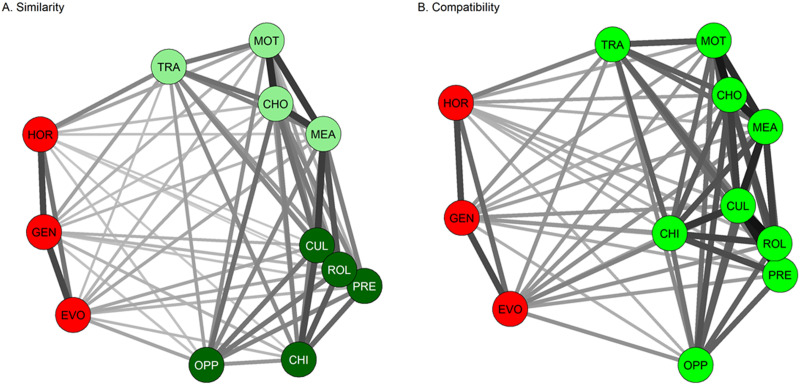
Visualization of multi-dimensional scaling and *k*-means clustering for (A) similarity and (B) compatibility of explanations with different framings, Study 1. Euclidean distance and line darkness represent similarity or compatibility. Networks have been subject to Procrustes rotation for comparability. Node colour represents cluster membership in *k*-means clustering. Abbreviations: HOR: hormonal; GEN: genetic; EVO: evolutionary; TRA: (psychological) trait; MOT: motivational; CHO: choice; MEA: meaning; CUL: culture; ROL: social roles; PRE: social pressure; CHI: childhood experience; OPP: opportunity.

The multidimensional scaling results for similarity are shown in Figure 2A. The stress metric was 0.21. This value is considerably less than the metric for randomly generated networks of this size, though there is no simple rule for what constitutes good fit of a multidimensional scaling solution (Mair et al., 2016). The optimal number of clusters was three. Cluster membership is indicated by colour-coding on Figure 2A. One cluster consisted of explanations that we can label ‘biological’ (genetic, hormonal, evolutionary); the second, ‘psychological’ states or processes (trait, choice, motivation, meaning); and the third cluster, explanations that would be described as ‘sociocultural’, though childhood experience was also included in this cluster (constituent members were culture, social role, social pressure, opportunity, and childhood experience).

The multidimensional scaling map for compatibility (Figure 2B, stress metric 0.20) was very similar to that for similarity (coefficient of congruence after Procrustes rotation, 0.98). The optimal number of clusters for compatibility was two rather than three. The clusters extracted for compatibility were identical to the ‘biological’ similarity cluster (genetic, hormonal and evolutionary), plus the other two similarity clusters combined. Within the large ‘non-biology’ cluster for compatibility, the relative distances of the explanations were much as for similarity, with the more psychological framings perceived as more compatible to one another than they were to the more sociocultural ones. However, culture and childhood in particular were rated as more compatible with ‘psychological’ explanations than they were similar to those explanations. This is why, in the compatibility clustering, the ‘psychological’ and ‘sociocultural’ clusters did not separate.

**Figure 3.**
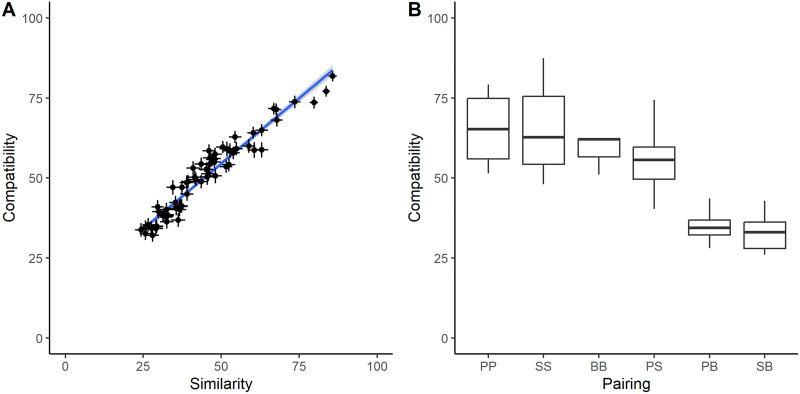
(A) Relationship between rated compatibility and rated similarity, Study 1. Each point represents a pair of explanatory framings. Horizontal and vertical error bars represent ± 1 standard error of the mean in the similarity and compatibility dimensions. (B) Boxplot of rated compatibility of pairs of explanations, by the clusters they respectively belong to in the similarity space, Study 1. PP: both ‘psychological’; SS: both ‘sociocultural’; BB: both ‘biological’; PS: one ‘psychological’ and one ‘sociocultural’; PB: one ‘psychological’ and one ‘biological’; SB: one ‘sociocultural’ and one ‘biological’.

The reason for the close congruence of the similarity and compatibility spaces shown in Figure 2 is that compatibility was treated, in our sample, as a near-synonym for similarity (Figure 3A). The correlation, across pairs of explanations, between their rated compatibility and their rated similarity was *r* = 0.96. Pairs of explanations belonging to different similarity clusters were perceived as much less compatible than pairs of explanations belonging to different similarity clusters (same clusters: mean compatibility 64.1, *SD* 11.8; different clusters: mean compatibility 42.7, *SD* 12.8; *t*(64) = −6.30, *p* < 0.001). More specifically, a pair of explanations was perceived as much more incompatible if one member belonged to the ‘biological’ similarity cluster and the other did not than in any other scenario (Figure 3B).

In general, our participants viewed explanations with different framings as neither wholly compatible nor wholly incompatible. Averaging across participants, the most compatible pair of framings (social role and social pressure) had a mean compatibility of 87.5, whilst the least compatible (hormonal and childhood) had a mean compatibility of 26.1. Of the 12,672 compatibility ratings given in total, 5677 (45%) were lower than the mid-point of the scale (i.e., more towards incompatible than compatible), with the remaining 6995 (55%) at or above the mid-point. There were 1114 (9%) ratings of 0 (i.e., the totally incompatible endpoint of the scale), and 727 (6%) of 100 (totally compatible). Of the 96 participants who rated for compatibility, 51 (53%) gave at least one rating of zero, and 28 (29%) gave at least 10 ratings of zero. All but 3 participants (97%) gave 10 or more compatibility ratings that were below the mid-point of the scale.

### Discussion

The main findings of Study 1 can be summarised as follows. In terms of similarity to one another, explanations fell into three clusters, a ‘biological’ cluster (framed in terms of hormones, genes and evolutionary advantage); a ‘psychological’ cluster (choice, meaning, motivation and psychological traits); and a ‘sociocultural’ cluster (culture, social role, social pressure, opportunity and childhood experience). The three clusters plausibly correspond to the domains of intuitive biology, intuitive psychology, and intuitive sociology respectively. Cluster membership seems to have been driven primarily by explanation content (does the explanation mention bodily or animal features; or mental states; or social networks and expectations that constrain the individual’s choices). Our results recover some of the higher-order dichotomies assumed in previous research, such as ‘biogenetic’ versus ‘psychological’ (Haslam & Kvaale, 2015), which corresponds to our biological versus psychological clusters; and ‘individualistic’ versus ‘societal’ (Cozzarelli et al., 2001), which corresponds to our psychological versus sociocultural clusters.

In terms of compatibility, ratings were typically around the middle of the scale, but with some pairs of explanations much less compatible than others. Compatibility and similarity judgements were strongly concordant: explanations were perceived as more compatible with one another exactly when they were perceived as more similar to one another. This is a non-obvious and possibly disturbing result from a scientific point of view. For example, a scientist might view a social role explanation and a hormonal explanation as dissimilar, but compatible (e.g., the hormonal changes provide the proximal pathway through which social expectations are internalised and expressed). It is therefore an important discovery that among these non-experts, dissimilar explanations are experienced as rather incompatible with one another. Since explanations with ‘biological’ framings were considered the most dissimilar from other kinds of explanation, they were also considered to be the most incompatible with other kinds of explanation. This cognitive clash may represent the different core procedures of intuitive biology from those of intuitive psychology and sociology (although, this is not the only possible explanation, see General Discussion).

A limitation of Study 1 is that similarity and compatibility judgements were made about particular tokens of explanations, in the context of specific behaviours. Generalizability to other kinds of behaviours and other tokens of explanation for them cannot be assumed. We aimed to minimize this concern by averaging across four different vignettes, and the similarity and compatibility judgements about the framings were similar in the four cases. However, judgements about causes can be influenced by what is perceived to be normal, and also by valence (Kirfel et al., 2022; Knobe & Samuels, 2013). Our behaviours would probably be considered to have negative (killing, teenage parenthood) or at best neutral (blood blessing) valence. Moreover, all of them would be considered uncommon or atypical (killing and teenage parenthood being atypical individual behaviours, and land diving and blood blessing being atypical group behaviours). The patterns could look different for cases of typical and/or positively valenced behaviours.

## STUDY 2

Study 1 described the mental map of explanation similarity in our study population. It suggested a three-way clustering of ‘biological’, ‘psychological’ and ‘sociocultural’ explanations. If these three clusters stem from the predominant activation of different core cognitive domains (intuitive biology, intuitive psychology and intuitive sociology), then people should make different patterns of inference according to which core domain the explanation activates most strongly. These inferences will follow the priors and principles of intuitive cognition in that domain. For example, intuitive biology characterizes organisms as owing their properties to an inner essence that is fixed and transmitted through reproduction (Atran, 1998; Linquist et al., 2011; Machery et al., 2019). Thus, to the extent that the explanation activates intuitive biology, people should infer fixity. There is prior evidence that this is the case. For example, when the explanation evokes a bodily basis, presumably activating intuitive biology, psychological disorders are seen as more immutable and transmissible (Berent & Platt, 2021a, 2021b; Haslam & Kvaale, 2015). Likewise, framing explanations for gender differences biologically makes people judge that the gender differences would be hard to change (Brescoll & LaFrance, 2004). Our general expectation is, therefore, that choosing one type of framing over another, without providing further specification, will trigger different inferences about the behaviour under description.

In Study 2, we used the same vignettes and explanations as in Study 1. Instead of asking participants to rate similarity and compatibility, we asked them to make judgements about what would be true of the behaviour if each of the 12 explanations were in fact correct. Specifically, we asked about *malleability* (how easily each behaviour could change); *externality* (the extent to which responsibility for the behaviour lies out in society rather than internally to individuals); and *simplicity* (whether the causes of the behaviour are simple or complex). As in Study 1, our aims were exploratory. We expected different explanations to have different profiles of malleability, simplicity and externality. We tested this explanation both by comparing ratings of every explanation to every other, and also by grouping the explanations into the similarity clusters discovered in Study 1, and examining how malleability, simplicity and externality vary between clusters.

### Methods

#### Pre-registration.

We pre-registered methods, materials and planned analyses at: https://osf.io/a7s45. Study 2 was conducted concurrently with Study 1 (although with different participants). Hence, the three-way similarity clustering of framings observed in Study 1 was not known. Analyses reported below that use the three clusters were therefore not pre-registered. There was also a pilot study for Study 2 whose results informed the design and are presented in the pre-registration of the main study.

#### Participants.

A total of 400 participants (200 male, 200 female; UK resident adults with first language English who did not participate in Study 1) were recruited via online recruitment platform Prolific (www.prolific.co). Mean age was 41.89 years (*SD* 13.28). Participants were mostly non-students (299 non-students, 25 students, 76 not stated), in employment (218 employed full or part-time, 83 not currently employed, 99 not stated). Participants received £2.20 for taking part.

For 10 surveys, the final submit button was not selected. The study contained a total of 12 attention checks (see below). 55 participants failed at least one of these and were excluded, leaving a final sample of 335.

#### Design.

We used the same four vignettes as Study 1, with approximately equal numbers of participants seeing each vignette. At the end of the vignette, respondents saw the list of 12 explanations that have been offered for the behaviour described in the vignette (the same list as in Study 1). Participants were then taken through each explanation in turn, in random order. Assuming the given explanation to be true, they were asked to indicate their agreement with six statements. Instructions made clear that participants were not rating their degree of belief that the explanation was true, but rather what would follow if the explanation were true.

#### Dependent Variables.

The six rating measures, all assessed on a 100-point slider anchored with ‘disagree’ and ‘agree’, were as follows.

1. This behaviour could change easily.
2. All of society is responsible for this behaviour.
3. This behaviour has a simple cause.
4. This behaviour is inevitable.
5. Responsibility for this behaviour lies within the individuals that do it.
6. The causes of this behaviour are complex.

These ratings were intended to give two-item measures of malleability (items 1 and 4), externality (2 and 5) and simplicity (3 and 6). We scaled each rating within subjects, as we were primarily interested in the within-subjects covariation of ratings across explanations, rather than the between-subjects covariation of ratings. Results were very similar without applying within-subjects scaling. We tested whether the six items covaried as intended by fitting a three-component PCA with oblimin rotation. The KMO statistic for the correlation matrix of the six items was 0.61, which is adequate for PCA but not excellent. The three components extracted were essentially uncorrelated with one another, and accounted respectively for 26%, 26% and 19% of the variation. The first component had loadings of 0.83 on item 1 and −0.82 on item 4. The second component had loadings of 0.90 on item 3 and −0.74 on item 6. The third component had loadings of 0.96 on item 2, but only −0.29 on item 5 (this item also loaded on both other components with 0.44 and 0.31 respectively). Thus, the pairs of items covary as intended for malleability and simplicity, but not well for externality, and the component for externality primarily reflects just one of the items. The scores from the PCA were used as the three dependent variables.

#### Attention Checks.

After completing the ratings for each explanation, respondents were shown one of the 12 explanations and asked (True/False) whether this was the explanation they just provided ratings about. Six of the attention checks show the right explanation (True), and six showed a different one (False).

#### Data Analysis.

As in Study 1, we pooled results across the four vignettes. Our first set of analyses used individual participant ratings as the unit of analysis. The within-subjects scaling removed the need for random intercepts by participant (since every participant had a mean rating of 0 with a standard deviation of 1). Using MANOVA and general linear models, we examined whether ratings differed by framing. For our second set of analyses, we calculated the mean malleability, externality and simplicity per framing; hence, the framing is the unit of analysis for these models. We grouped the framings into the similarity clusters observed in Study 1. We used MANOVA and linear mixed models (with a random effect of framing) to examine whether the mean malleability, externality and simplicity differed by framing cluster.

### Results

The 12 framings received significantly different ratings from one another across the set of three dependent variables (*F*(33, 11550) = 108.64, *p* < 0.001). Specifically, the framings differed significantly from one another in terms of malleability (*F*(11, 3850) = 160.62, *p* < 0.001), externality (*F*(11, 3850) = 148.26, *p* < 0.001)) and simplicity (*F*(11, 3850) = 61.93, *p* < 0.001). Framings belong to different clusters (from Study 1) received different average ratings across the set of three dependent variables (*F*(2, 9) = 9.06, *p* = 0.007); specifically, they received significantly different average ratings for malleability (*F*(2, 9) = 7.02, *p* = 0.015), externality (*F*(2, 9) = 14.29, *p* = 0.002) and simplicity (*F*(2, 9) = 5.25, *p* = 0.031).

**Figure 4.**
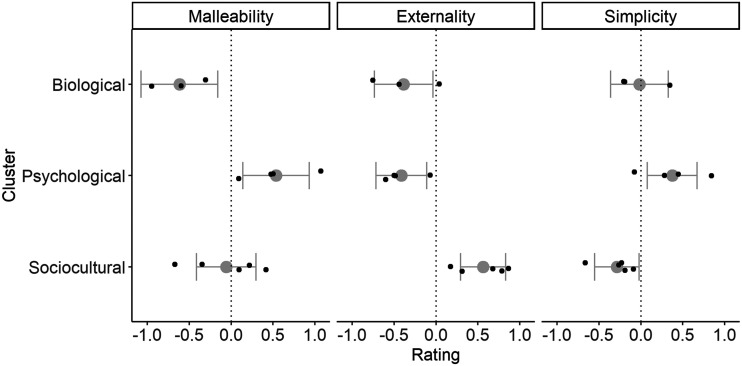
Ratings of malleability, externality and simplicity across framing clusters, Study 2. Grey points and whiskers show estimated marginal mean and 95% confidence interval at the cluster level. Superimposed black points represent means for individual explanations. Note that since variables are scaled, zero always represents the mean across all explanations.

Figure 4 visualizes these results. Explanations belonging to the ‘biological’ cluster were rated as relatively unmalleable; those belonging to the ‘psychological’ cluster as relatively malleable; and those belonging to the ‘sociocultural’ cluster generally intermediate. There was also variation within clusters. For example, within the ‘biological’ cluster, genetic was the least malleable, and hormonal the most; within ‘psychological’, choice was extremely malleable and meaning sat closer to the mean for ‘sociocultural’ explanations; and within ‘sociocultural’, opportunity was fairly malleable whilst culture was very non-malleable. As for externality, the ‘sociocultural’ explanations were relatively external, whereas the ‘biological’ and ‘psychological’ framings were relatively internal. In the case of simplicity, differences across clusters were the least marked, but ‘psychological’ explanations were the simplest on average, and ‘sociocultural’ the least simple.

### Discussion

Different framings provoked different judgments in our participants. Sociocultural explanations were perceived as locating the causality out in society rather than within the individual, whereas both biological and psychological explanations were rated as locating causality within individuals. Partial exceptions to this generalization were evolutionary explanations and explanations in terms of meaning, which were perceived as intermediate between internal and external. These findings suggest our participants did attend to the material. There is an obvious sense in which psychological and biological explanations do concern intra-personal processes, and in which evolutionary and meaning explanations are atypical. Evolutionary explanations concern the fit between organism and environment, and explanations in terms of meaning concern processes that are internal to the mind but also shared with others in social networks. We also found differences in how simple the explanations were perceived as being: psychological trait, motivational, choice and hormonal explanations were perceived as relatively simple. All other explanations were perceived as complex compared to these.

The finding with the most important implications is that explanations with different framings were perceived to imply different levels of malleability of the behaviour. Making reference to biology, in the absence of any other information, triggered the inference that the behaviour would be hard to change. Whilst this inference might be correct for certain cases of genetic explanation, it need not be true for all biological explanations. For example, hormone levels are highly dynamic, and can change rapidly in response to environmental inputs. Moreover, adaptive evolutionary explanations really concern the consequences of the behaviour for survival and reproduction. How rapidly the behaviour might change if the context changes will depend on the proximal mechanisms that deliver the adaptive behaviour. Thus, merely demonstrating that there is a ‘biological’ basis does not necessarily mean that the behaviour could not change easily. However, for our participants, all ‘biological’ explanations led to the inference of below-average malleability. Possibly this is due to our ‘biological’ explanations cueing intuitive biological cognition, under whose logic creatures follow inner, immutable, transmissible essences (Atran, 1998). For this reason, actors who find implications of non-malleability congenial may be particularly drawn to transmitting explanations with biological framings (as Brescoll and LaFrance (2004) found for politically conservative newspapers and research on gender differences). However, it is a hazard for any researcher who is interested in biological mechanisms or evolutionary adaptiveness, but does not believe, or wish to be taken as implying, that the behaviour is not malleable (see also Dar-Nimrod & Heine, 2011).

By contrast, ‘psychological’ explanations (especially psychological traits, motivation, and choice) produced the highest inferred malleability. This may stem from their engagement of intuitive psychology, whose function is to predict and intervene in individuals’ behaviour as time and context changes (Ho et al., 2022). In line with this function, intuitive psychology sees behaviour as stemming from transient and reversible inner states such as beliefs and desires. The researcher who frames their results at this level of the causal chain may thus be taken to imply—whether they will it or no—that changing beliefs and desires is relatively easy and sufficient to produce behavioural change. However, this will not necessarily be the case if individual agency is heavily constrained by social-structural factors (Chater & Loewenstein, 2023; Haslanger, 2016; Ross, 2023; Vasilyeva & Lombrozo, 2020).

The sociocultural explanations were markedly heterogeneous in terms of inferred malleability. Explanations in terms of opportunity produced fairly high ratings of malleability. Explanations in terms of culture, on the other hand, led to the inference that behaviour change was almost as difficult as in cases of genes and evolution. The difference between genes and culture in the minds of our participants was not that they implied different levels of malleability—these were about the same—but that genes locate the causality within the individual, and culture locates it out in society. These findings are interesting in light of the way culture is often described in academic literature, as an inheritance system at least partly analogous to genes, with a fair degree of historical persistence. It also relates to critiques of the use of the culture concept (for example, in the case of ‘the culture of poverty’, Black and Dolgon (2021)). These critiques see the term as essentializing the current behaviours of people in poverty, obscuring the extent to which those behaviours may in some cases be dynamic, rational, responses to very specific structural factors or policy measures.

## STUDY 3A

Studies 1 and 2 found evidence that people group explanations of behaviour into ‘biological’, ‘psychological’ and ‘sociocultural’ clusters. Further, people assume differential malleability according to which framing is invoked. Study 3 (A and B) extends Studies 1 and 2 by focusing on how the choice of framing affects the preferred choice of intervention. That is, what kinds of measures are plausible candidates for making the behaviour change? There are close connections between explanations (why did X happen?), counterfactuals (what would have to have gone differently for X not to happen?), and intervention strategies (how can X be prevented from happening?) (Gerstenberg et al., 2017; Halpern & Pearl, 2005; Hitchcock & Knobe, 2009; Quillien, 2020; Woodward, 2003). Pragmatically, the fact that someone has adopted a particular framing suggests that they think it is the optimally relevant one, and therefore that the most promising intervention strategies reside at this level (Hilton, 1990).

We therefore expect the choice of framing to guide cognition about effective interventions. In communication studies, one of the functions attributed to framing is to guide the search for remedies (Entman, 1993). Previous research suggests that there are effects of this kind. Physicians intuit that pharmacological treatment will be more effective for disorders they consider biogenetic in origin, and talking cures for disorders they consider of psychological origin (Ahn et al., 2009; Marsh & Romano, 2016). Adopting a ‘biological’ framing might not just increase the perceived effectiveness of pharmacological interventions, but also decrease the perceived effectiveness of other kinds of interventions, such as political or socioeconomic reform. The reason for this would be that if the speaker had believed those kinds of interventions to be the right ones, she would have framed the explanation in a different way (Hilton, 1990).

In Study 3A, we used two of the vignettes presented in Studies 1 and 2, with slight wording modifications. In a between-subjects design, participants saw the description of the behaviour, plus one explanation, or no explanation. They were then asked how much difference could be made to the behaviour by changing biology (for example through drugs), changing psychology (for example through thinking training), and changing society (for example through political reform). Since every participant was asked about the effectiveness of all three types of intervention, the design potentially allowed us to detect multiple effect patterns. For example, an explanation with a certain framing might be perceived as making all interventions more effective (relative to no explanation), all interventions less effective, or some interventions more effective and some interventions less so.

We pre-registered three predictions (see pre-registration link below), using the explanation cluster (rather than the individual explanation) as the explanatory variable:

P1. The cluster of explanation offered will affect the rated effectiveness of the three types of interventions.
P2. Offering an explanation belonging to a particular cluster will *increase* the rated effectiveness of interventions belonging to that cluster (relative to ‘no explanation’).
P3. Offering an explanation belonging to a particular cluster will *decrease* the rated effectiveness of interventions belonging to the other two clusters (relative to ‘no explanation’).

We also performed exploratory analyses at the individual explanation level rather than the level of the explanation cluster. Participants of Study 3A who were in the ‘no explanation’ group additionally completed Study 3B (see below).

### Methods

#### Pre-registration.

We pre-registered methods, materials, and planned analyses at: https://osf.io/aqsz3.

#### Participants.

Participants were recruited via Prolific as per Studies 1 and 2. We pre-registered a sample size of 320. However, due to an error, the participants in the ‘no explanation’ condition were not shown the Study 3B questions (for Study 3B, see below), leading us to add 80 more participants in the ‘no explanation’ condition. Preliminary inspection of the data after the 320 + 80 participants suggested we were under-powered to determine what was happening at the individual explanation level. We therefore added another 320 participants as per the original design. Post-hoc increases in sample size inflate type-I error rates. We therefore recalculated the critical *p*-values required in the final sample to keep overall type-I error rates at 0.05, using the *p*_*crit*_ formula (Sagarin et al., 2014), for one additional round of data collection, and assuming that any *p*-value less than 0.2 would have led us to collect more data. This corrected *α* was 0.04, which should therefore be considered the appropriate *α* for the results presented below.

#### Design.

We used vignettes from Studies 1 and 2, homicide and teenage parenthood. Each participant saw one vignette. In Study 1, there were unequal numbers of framings in the three clusters: three ‘biological’, four ‘psychological’ and five ‘sociocultural’. To equalize the representation of clusters, we added an additional biological framing (‘brain circuits’), and omitted the ‘childhood’ explanation (from ‘sociocultural’), producing four framings per cluster. The probability of being assigned to the ‘no explanation’ condition was four times that for every other condition. This is so that when explanations are aggregated to the cluster level, the numbers of observations in the ‘no explanation’, ‘biological’, ‘psychological’ and ‘sociocultural’ clusters were approximately equal (see Table 1 for achieved sample sizes).

**Table 1.** Design summary, with realised numbers of participants in each cell.

Vignette	No explanation	Biological		Psychological		Sociocultural	
Homicide	122	Brain	27	Choice	15	Culture	20
		Evolutionary	23	Meaning	16	Opportunity	21
		Genetic	20	Motivation	14	Pressure	17
		Hormonal	18	Trait	25	Role	21
Teenage parenthood	115	Brain	22	Choice	23	Culture	16
		Evolutionary	17	Meaning	20	Opportunity	21
		Genetic	19	Motivation	23	Pressure	24
		Hormonal	20	Trait	22	Role	19

#### Vignettes.

The vignettes describe the behaviour (homicide or teenage parenthood respectively) followed by the statement: ‘scientists have discovered that …’ and one of the explanations (for example, “In the town of XXXX, young men commit a large number of murders each year. Scientists have discovered that young men in XXXX kill because of low levels of activity in a particular brain region”). The vignettes and explanations had slight wording differences from Studies 1 and 2, particularly to make all options more grammatically similar and all contain an explicitly causal ‘because’ (see pre-registration at https://osf.io/8ba5m for details). In the no-explanation condition, we omitted the ‘scientists have discovered that …’ statement.

#### Dependent Variables.

After reading each vignette and any explanation, participants responded by answering three questions on a 100-point scale anchored with ‘none’ and ‘a great deal’. The wording for the homicide vignette was:

- How much could the rate of homicide be decreased by changing biology, for example through tablets, injections, or brain stimulation?
- How much could the rate of homicide be decreased by changing psychology, for example through education, persuasion, or thinking training?
- How much could the rate of homicide be decreased by changing society, for example through better political representation, increasing incomes, or better jobs?

The wording for the teenage parenthood vignette replaced the words ‘the rate of homicide’ in the question with ‘the rate of teenage parenthood’).

#### Predictions and Analysis Plan.

Pre-registered predictions were as listed in the Study 3 introduction. In the pre-registration, we acknowledged the possibility that P2 and P3 might hold for some types of framing but not others. For example, biologically-framed explanations might reduce the rated effectiveness of societal interventions, but not vice versa. We made no a priori predictions about this heterogeneity.

**Figure 5.**
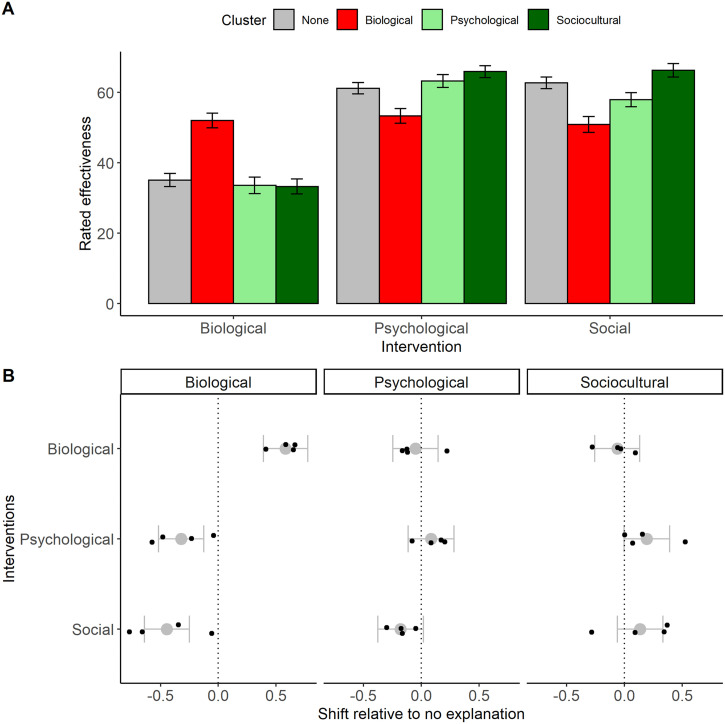
(A) Mean rated effectiveness of different interventions (*x* axis) after explanatory framings belonging to different clusters (colours) have been offered, Study 3A. Error bars represent ± 1 standard error of the mean. (B) Effect of providing a framing of a particular cluster (columns) on the rated effectiveness of a particular type of intervention (rows), relative to ‘no explanation’, Study 3A. Grey points and whiskers show standardized effect sizes and their 95% confidence intervals at the explanation cluster level. Superimposed black points are the standardized effect sizes when the individual framing is used as the unit of analysis.

We fitted linear mixed models with the predictors: intervention type, framing cluster, and their interaction; and rated effectiveness as the outcome. P1 implies effects of framing cluster, either as main effects or interactions with intervention type. P2 and P3 imply significant interactions between intervention type and framing cluster. We included random effects of individual framing, and participant. We also ran the same analyses with individual framing as the independent variable, and these results are superimposed on Figure 5. Data from the two vignettes were pooled.

### Results

In a mixed model with effectiveness as the outcome and intervention, framing cluster, and their interaction as predictors, there was a significant effect of intervention (*F*(2, 1431.77) = 207.35, *p* < 0.001). This was driven by an overall endorsement of psychological and social interventions as more effective than biological ones (estimated marginal means: biological, 38.5 (*SE* 1.55); psychological 60.9 (1.55); social 59.4 (1.55); see Figure 5A). The main effect of framing cluster was not significant (*F*(3, 6.43) = 0.44, *p* = 0.73), but there was a significant interaction between intervention and framing cluster (*F*(6, 1431.74) = 23.33, *p* < 0.001).

To examine this interaction, we broke the data down into the three types of intervention. We ran separate models to examine the effect of each type of framing (relative to the reference category of ‘no explanation’) on rated effectiveness for that type of intervention. Results are shown in Figure 5B. Providing a biologically-framed explanation both increased the rated effectiveness of biological interventions, relative to no explanation, and decreased the rated effectiveness of psychological and social interventions, relative to no explanation. By contrast, providing a psychologically- or socially-framed explanation had no systematic effect of the rated effectiveness of any type of intervention. Thus, we found support for P1 (choice of explanation cluster affects rated effectiveness of interventions) in general, but P2 (framings enhance rated effectiveness of congruent interventions) and P3 (framings suppress rated effectiveness of incongruent framings) were only supported for ‘biological’ framings.

### Discussion

Study 3A confirmed that the choice of explanation can matter for inferences about what kinds of interventions are potentially effective for changing the outcome. However, it was only biological explanations that had clear implications for intervention effectiveness: providing a biologically-framed explanation both *enhanced* the perceived effectiveness of biological interventions such as drugs; and, importantly, *suppressed* the perceived effectiveness of psychological and social interventions. These findings demonstrate the competitive nature of framings (Chater & Loewenstein, 2023): adopting a framing both facilitates cognition about interventions at the level of that framing, and discounts interventions at other levels.

The lack of either enhancement or suppression effects when we provided psychological or sociocultural-type framings is perhaps puzzling. One possibility is that these are our participants’ default-type explanations for the kinds of behaviours in our vignettes. Thus, providing explanations of these types did not change anything for our participants compared to providing no explanation. Compatible with this possibility, rated effectiveness of social and psychological interventions was higher overall than those of biological interventions, including in the ‘no explanation’ condition. Providing a ‘biological’ explanation may have caused the pragmatic inference that the default non-biological explanations did not apply here, making it quite natural to assume that their associated interventions did not work either. By contrast, providing an explanation with a ‘psychological’ or ‘sociocultural’ framing just confirmed assumptions people already held, including their prior assumptions about relevant ways of intervening.

## STUDY 3B

Study 3A showed there can be competition between explanations with different framings, and that framings are linked to assumptions about what kinds of interventions will make a difference: being given a biologically-framed explanation both increased the perceived effectiveness of a biological intervention, and reduced the perceived effectiveness of psychological and social interventions. Study 3B was an ancillary study, added to the questions of the Study 3A participants in the ‘no explanation’ condition, that tested a corollary of these findings. If ‘biological’ and ‘non-biological’ explanations are perceived to stand in competitive exclusion, then the same might be true for interventions. That is, participants who learn that a biological intervention is effective in changing the behaviour might infer that other kinds of intervention will have no effect, whilst those who learn that a ‘biological’ intervention is ineffective at changing the behaviour might infer the opposite, that another interventional approach is required.

In Study 3B, after participants had read the vignette and rated biological, psychological and social interventions for changing the behaviour, we gave them additional information that one of the intervention types had been tried and either had a big effect on the behaviour (effective condition) or had no effect on the behaviour (ineffective condition, between subjects). We then asked participants to rate whether they now thought the other two intervention types would be less effective than they previously estimated, more effective, or the same. We predicted that learning that a biological intervention is effective would reduce rated effectiveness of other kinds of interventions, and learning that a biological intervention is ineffective would increase rated effectiveness of other kinds of interventions.

### Methods

#### Pre-registration.

We pre-registered methods, materials, and planned analyses along with Study 3 at: https://osf.io/aqsz3.

#### Participants.

Participants from the ‘no explanation’ condition of Study 3 (*n* = 153).

#### Design.

Study 3B was a between-subjects three (intervention type) by two (effective or ineffective) factorial design, with each participant reporting the dependent measure twice (once for each of the two intervention types other than the one mentioned).

#### Stimulus.

After completing the dependent measures of Study 3A, Study 3B participants were given the following information.

Scientists have discovered that [changing biology, for example through drugs/changing psychology, for example through thinking training/changing society, for example through better political representation] [makes no difference/substantially reduces] [the rate of homicide/the rate of teenage parenthood].

#### Dependent Measure.

Participants were then asked:

Does the information given above make you think that [changing psychology/changing biology/changing society] would be less effective in changing [the rate of homicide/the rate of teenage parenthood] than you previously thought, more effective, or no change?

Two questions were displayed, for the two intervention types other than the one mentioned in the stimulus. Participants responded on a slider from ‘less effective’ to ‘more effective’, with ‘no change’ at the mid-point.

#### Predictions and Analysis Plan.

We pre-registered two predictions:

P1. Learning that one class of intervention is ineffective will increase belief in the effectiveness of the other two classes of intervention.
P2. Learning that one class of intervention is effective will decrease belief in the effectiveness of the other two classes of intervention.We specified that P1 and P2 might hold for some combinations of intervention classes but not others. We tested P1 and P2 with linear mixed models with intervention mentioned, intervention asked about, and effectiveness condition (effective or ineffective) as the predictors. We also used one-sample t-tests to examine whether average ratings deviated from ‘no change’ for particular combinations of intervention mentioned and intervention asked about.

### Results

**Figure 6.**
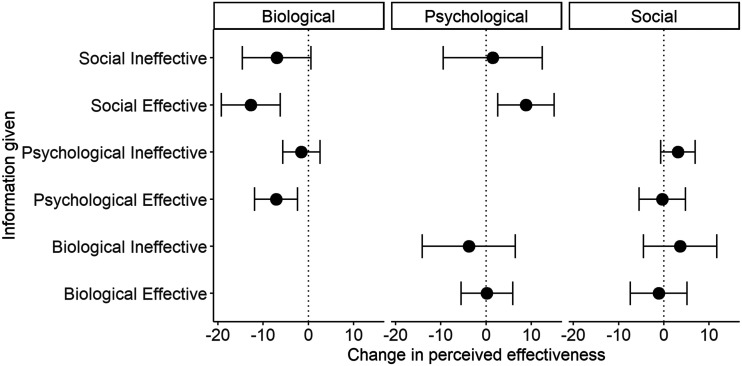
Study 3B results. Change in perceived effectiveness of an intervention (columns) by which other intervention had been mentioned, and whether it had been described as effective or ineffective (rows). Points show the mean shift and whiskers the 95% confidence interval. Where the 95% confidence interval does not include zero, that category of information significantly shifts average perceived effectiveness.

We fitted a linear mixed model with change in perceived effectiveness as the outcome variable, and predictors of intervention in question, intervention mentioned, and whether the mentioned intervention was described as effective or ineffective. There was a significant interaction between intervention in question and intervention mentioned (*F*(1, 147) = 4.30, *p* = 0.04). All other interactions were non-significant (*p* values > 0.05). This suggests that learning about the effectiveness or ineffectiveness of some interventions affects the perceived effectiveness of some other interventions. To probe which ones affected which, we calculated the mean and 95% confidence interval of change in effectiveness for each combination of intervention in question, intervention mentioned, and effectiveness or ineffectiveness (Figure 6). Where the 95% confidence interval does not include zero, we can conclude that this class of information significantly changes perceived effectiveness of that class of intervention. As Figure 6 shows, learning that a psychological or social intervention is effective reduced the perceived effectiveness of biological interventions. Curiously, learning that a psychological or social intervention was ineffective did not increase the perceived effectiveness of ‘biological’ interventions. Nor did learning that a biological intervention was effective reduced the perceived effectiveness of psychological or social interventions. The only other significant shift was that learning that a social intervention was effective increased the perceived effectiveness of a psychological intervention. The converse did not hold.

### Discussion

If different explanations suggest the effectiveness of different types of intervention, and different types of explanation compete with one another, we reasoned that learning that one type of intervention does (or does not) work might generate the inference that another type of intervention will not (or will) do so. We found only partial evidence for such effects. Specifically, learning that a social or psychological intervention is effective led participants to reduce their perceived effectiveness of a biological intervention. The converse did not hold: learning that a biological intervention was effective did not reduce the perceived effectiveness of psychological or social interventions. On the face of it, this is puzzling: a widespread concern in the literature on psychological disorders, for example, is the concern that publicity around pharmaceutical treatments leads sufferers, clinicians and the general public to neglect the promise of addressing their prevalence through psychological and particularly socioeconomic means (Davies, 2021). Our results do not concur with this, though they do suggest the converse effect: stressing the potential effectiveness of political and social approaches to psychological suffering would undermine the perception of effectiveness of drugs. A possible explanation is that, for the particular behaviours described in our vignettes, our participants thought that psychological and social interventions were much more effective than biological ones at baseline (see Study 3 results). Thus, learning that an intervention-class they regarded as weaker (biological) was effective was not sufficient to diminish their belief in interventions they already regarded as stronger; whereas learning that interventions of their favoured or default class were indeed effective further inhibited the perceived effectiveness of the disfavoured class.

Learning that psychological and social interventions were *in*effective did not increase the perceived effectiveness of biological interventions. If anything, it tended towards decreasing it. A possible explanation is the ineffectiveness of psychological and social interventions is a cue that the behaviour is completely non-malleable. This is particularly true given that our participants thought the psychological and social interventions to be the strongest available at baseline. Thus, if even those do not work, it seems unlikely that a class of intervention perceived to be weaker in the first place (biological) would do so. Hence, learning about effectiveness (the behaviour is malleable, and responds to our best interventions) would not necessarily have the mirror image of the consequences of learning about ineffectiveness (the behaviour is not malleable even with our best interventions). We also found that learning about the effectiveness of a social intervention *increased* the perceived effectiveness of a psychological one, presumably because it provided evidence of malleability in general.

In summary, Study 3B found some evidence that knowing that one class of intervention is effective can undermine perceived potential of other kinds of intervention. Specifically, this phenomenon only occurred across the biological/non-biological boundary: the effectiveness of psychological and social measures undermined the perceived effectiveness of biological measures like drugs, although the converse, did not hold.

## GENERAL DISCUSSION

These studies have charted the mental maps of different framings held by our participants, and begun to probe the inferences they make from the choice of one framing over another. Although all three studies had pre-registered predictions and designs, we describe this work as primarily exploratory rather than testing a strong theory or single causal model. We remind the reader that our participants were not professional academics and mostly not students. Although not a representative sample of the UK population, their responses are likely to be a reasonable guide to how different types of explanation are understood in the non-expert UK population at large.

### Key Findings and Their Relation to Existing Literature

Our participants perceived family resemblance between certain kinds of explanations. Specifically, they produced, unguided by us, ‘biological’, ‘psychological’ and ‘sociocultural’ clusters of explanations (Study 1). They rated the constituent explanations of each of these groupings to be relatively similar to one another, and more different from the members of other groupings. These clusters do map onto groupings of explanations that have been proposed in the literature before. For example, our biological and psychological clusters map respectively onto the ‘biogenetic’ and ‘psychological’ of Haslam and Kvaale (2015), and our sociocultural cluster appears to correspond to social-structural explanation (in the sense explored by Vasilyeva et al., 2018; Vasilyeva & Lombrozo, 2020). The difference is that our methodology in Study 1 allowed these groupings to emerge from participant responses, rather than us assuming their existence a priori.

Our participants were certainly not complete ‘incompatibilists’. That is, in Study 1, they perceived many cases where, given the truth of an explanation with one framing, it was possible at least to some extent for an explanation with a different framing also to be true. However, we were struck by the substantial degree of incompatibility that they perceived amongst certain combinations of explanations. In particular, the more dissimilar they perceived two explanations to be, the more incompatible they felt them to be. As biological explanations were perceived as the most dissimilar from others, that meant they were also rated as the most incompatible with others. Thus, it seems that the non-expert conception of whether two explanations are compatible is simply whether they seem similar. We return later in the discussion to how this departs from the scientific conception of compatibility. We note here that it gives rise to the possibility of ‘framing discounting’, similar to the explanation discounting that has been studied elsewhere (Heussen, 2010; Kelley, 1973; Sloman, 1994). That is, the acceptance or availability of an explanation with one framing has the potential to make explanations with different framings less plausible or compelling.

Provided with an explanation, our participants made corresponding inferences about the locus of causation of the behaviour, and about malleability (Study 2). Both ‘biological’ and ‘psychological’ explanations led participants to infer that the causation is internal to the individual rather than external. However, ‘biological’ explanations were taken to imply that the behaviour is not malleable, whereas ‘psychological’ explanations implied a high degree of malleability. This finding echoes findings in the literatures on gender differences, and psychological disorders (among other topics, see Dar-Nimrod & Heine, 2011). Framing gender differences biologically produces the inference that they are relatively immutable, compared to framing them socially (Brescoll & LaFrance, 2004). A ‘biogenetic’ conceptualization of psychological difficulties encourages the view that those disorders are essential features of their sufferers, and not easy to change (Berent & Platt, 2021a; Haslam & Kvaale, 2015; Lebowitz et al., 2013). On the other hand, ‘psychological’ framings are sometimes ‘cruelly optimistic’: they seem to imply that change is easier than it actually is, especially when persistent socio-structural factors are integral to the actual network of causes (Brickman et al., 1982; Chater & Loewenstein, 2023).

Study 3 provided some evidence for an intuitive principle that ‘the medicine must fit the cause’. That is, the interventions that seem most likely to be effective are congruent to the framing of the causes of the behaviour. Specifically, providing a ‘biological’ explanation for a phenomenon both boosted the perceived effectiveness of intervening biologically, such as through drugs, and reduced the perceived effectiveness of psychological and societal change (Study 3A). Moreover, providing evidence that psychological or societal interventions were effective reduced the perceived likelihood of a biological intervention working (Study 3B). These effects are readily interpreted in the light of the literature on the cognitive function of causal explanation. Since causal explanations guide the search for ways of acting to make the world different (Quillien, 2020; Woodward, 2003), framing the causes in a certain way will naturally activate the search for interventions at the level of that framing.

### Origins of Observed Patterns

Having studied just one population, we cannot say how widespread or robust these cognitive patterns are. They could represent no more than the influence of a particular set of discursive practices, cultural and religious histories, or educational practices in the UK. However, it is worth investigating the hypothesis that they represent more recurrent and reliably developing features of human minds in general. In particular, we suggest that the key findings might be explained by rooting them in two bodies of existing work: core cognition (e.g., Spelke & Kinzler, 2007) and relevance theory (Sperber & Wilson, 1995).

Human core knowledge embodies domain-specific principles and priors. These are different for the domains of biology, psychology, and social relations, giving rise to distinct intuitive theories in each of these areas (Spelke & Kinzler, 2007). Activation of one these theories will feel different from activation of the others, and possibly incommensurate, giving rise to the perception of incompatibility. Different intuitive theories have different operating principles. For example, positing fixed and typical essences is central to intuitive biology (Atran, 1998; Linquist et al., 2011; Machery et al., 2019), whereas tracking and changing transient mental states is central to intuitive psychology (Ho et al., 2022). The different inferences about malleability when a biological versus a psychological explanation is offered may follow directly from these different operating principles.

Domain-specific intuitive theories interact with pragmatic principles when explanations are framed. When a speaker offers an explanation, they are aiming not just to utter a true causal statement, but to draw the hearer’s attention to the kinds of actions that could have most easily made a difference to the outcome (Hilton, 1990; Quillien, 2020). The default intuitive theory to bring to bear on human behaviour is probably intuitive psychology, and the default assumption for human competences appears to be that they are malleable (Wang & Feigenson, 2019). If a speaker chooses a different framing (for example, a genetic or neuroscientific one), the hearer can use pragmatic principles (Sperber & Wilson, 1995) to infer that this is because that speaker believes that the default framings do not work well in this case, and is guiding the hearer to look elsewhere. Hence, the speaker’s mere adoption of a framing can be taken as some evidence that they believe the most obvious assumptions (e.g., of malleability) do not apply, and the most obvious interventions (e.g., social or psychological ones) would not be good. This could explain the effects in Study 3A, where the adoption of a ‘biological’ explanation both enhanced the perceived efficacy of interventions like drugs, and suppressed the perceived efficacy or social or psychological interventions.

The claims of this section—that the patterns we observe arise from the structure of core cognition, combined with pragmatic communicative principles—are speculative. To test them would require additional kinds of data. There are already bodies of research on the emergence of distinctively biological, psychological, and sociological modes of thought in childhood (Thomas et al., 2022; Wellman & Gelman, 1992), and these could be extended to thought about compatibility, malleability, and interventions. Likewise, the cross-cultural generality of these patterns should be investigated. A possible model is the recent work on the distinction between psychological experience (‘mind’) and bodily sensation (‘body’) in different cultures (Weisman et al., 2021). The authors found a robust distinction between properties of minds and properties of bodies across adults and children in five cultures, but also subtle differences between cultures in where ambiguous properties like emotion were placed.

### Accuracy and Implications for Scientific Communication

We have conceptualized the compatibility and malleability findings as arising from pre-scientific intuitive theories. Does this mean that our participants’ judgements were wrong? The present case is not comparable to the study of intuitive theories of motion, where there is, for each stimulus, an actual motion path to which participants’ intuitions can be compared (McCloskey et al., 1980). Our vignettes and explanations are fictional, so the true answers are not defined. We cannot therefore show that our participants erred. Indeed, for everyday purposes, it might work well to divide the world into some phenomena that need to be thought of biologically, some psychologically, and some structurally, and have different priors about each of these classes. However, there are many complex phenomena where scientists wish to pursue the causal processes at multiple levels. Our findings suggest characteristic ways that science may be misunderstood.

First, participants treated all ‘biological’ explanations (genes, hormones, evolution) as largely interchangeable and with similar implications for malleability and interventions. But not all ‘biological’ explanations are alike. Positing a neural or hormonal basis for a behaviour does not in any way imply the belief in a genetic basis. Previous literature has also shown that hearers infer genetic transmissibility from any mention of a proximal neural mechanism (Berent & Platt, 2021a). This is logically a non-sequitur: a researcher could coherently believe that depression has a characteristic neural mechanism, and yet is still distally caused by, for example, poverty, and not genetically heritable. Our findings suggest that researchers who frame their explanations in terms of one type of ‘biology’ (adaptive consequences, neural substrate) could often find their claims mistakenly assumed to be about some of other type of ‘biology’ (e.g., genetic heredity).

Second, ‘hybrid’ or ‘multilevel’ accounts of causality will be hard for people to understand, and may often be misunderstood. For example, the evolutionary psychology paradigm characterizes human behaviour as arising from the interaction between current social environments and flexible evolved cognitive mechanisms (Tooby & Cosmides, 1989). However, it is often mischaracterized, even in the scholarly literature and by some adherents, as claiming that human behaviour is innate and inflexible (‘hard-wired’) and the current environment causally unimportant (Frankenhuis et al., 2013; Nettle & Scott-Phillips, 2023). Likewise, there is a literature on the effects of socioeconomic disadvantage on the developing brain (Hyde et al., 2022). This is integrative research that pursues causal chains from their social-structural origins through to proximal neural mechanisms, via the psychological mediating variables involved in parenting. Contributors to this literature encounter two hazards. On the one hand, they have been accused of genetic determinism, even eugenicism, because they are taken to be positing some inherent and immutable biological deficiency in people who live in poverty (Tolwinski, 2019). On the other, they have been accused of blaming impoverished parents for not taking good enough care of their children, when the real culprits are structural (Hyde et al., 2022). That is, the researchers are truly studying social-structural factors and their consequences, but because they measure biological variables like brains, they are misunderstood as making claims about non-malleable, heritable essences; and because they measure psychological variables such as caregiving, they are misunderstood as saying that low-income parents just need to behave differently. The researchers counter these misunderstandings with an active strategy, ‘plasticity talk’ (Tolwinski, 2019). This is the deliberate and repeated insistence that the brain, although a biological organ, is malleable and is both directly and indirectly influenced by what is going on in society.

The idea of ‘plasticity talk’ leads us to our final point about scientific communication. Our findings suggest that pluralism about causes (causes can be identified at multiple compatible levels), and pluralism about interventions (being able to identify a substrate at a biological level does not mean the only possible intervention is at that level) are not completely intuitively obvious. However, people can presumably appreciate them if they are actively and clearly pointed out. This, we contend, explains the continued market for papers and books that set out ways of integrating multiple levels of causality, and avoiding fruitless dichotomies such as immutable nature versus formless nurture (Mameli & Bateson, 2011; Marr, 1982; Nettle, 2009; Tinbergen, 1963). That is, active ‘plasticity talk’ and ‘plurality talk’ continue to be required, in order to counteract the cognitive attractors formed by the separateness of intuitive biological, psychological and sociocultural clusters. We would recommend any researchers whose work spans causal levels, or seeks internal substrate of structural causes, to actively anticipate likely misunderstandings and use appropriate pre-emptive talk.

### Societal Implications: Framing Contests

If framings partially compete with one another, and guide the cognitive search for interventions, it is in the interests of any party favouring a certain class of intervention to advocate the framing that suits their interests. Such ‘framing contests’ have long been studied in communication studies (Dan & Raupp, 2018; Entman, 1993). For example, more conservative newspapers, in reporting research on gender differences, privilege biological explanations, because these suggest non-malleability of traditional roles (Brescoll & LaFrance, 2004). The food industry prefers to frame obesity in terms of psychological traits or lack of knowledge, which suggests interventions to inform and educate consumers (Jenkin et al., 2011). Adopting this framing rather than a social-structural one pre-empts reflection on regulation and taxation interventions that might be unwelcome to these companies. Pharmaceutical companies strongly promote the framing of psychological problems as ‘diseases of the brain’, which naturally privileges drug intervention (Davies, 2021; Moncrieff, 2007; Read & Cain, 2013). Chater and Loewenstein (2023) argue that behavioural public policy, by focusing on individual-level ‘nudges’, has become effectively complicit with interests that want to avoid social-structural reform (though for a rebuttal of this argument, see Hallsworth, 2023).

The existence of the literatures discussed in the previous paragraph shows that controlling the framing is often an important goal, since it subtly directs the discourse on what if anything could be done. The resistance encountered by brain researchers who study the consequences of socioeconomic disadvantage is presumably not due to activists literally disbelieving that poverty could affect the brain. Instead it seems to be attributable to the fear that, the moment we are foregrounding brains, we are no longer foregrounding structural inequity (Tolwinski, 2019). The ‘plasticity talk’ used by these researchers is employed to show, counterintuitively, it is *by* talking about brains that we can demonstrate just how serious structural inequity is.

### Malleability

We conclude with a final question: how easily can the cognitive patterns we have described can be overcome. Formal training in the social and behavioural sciences involves teaching ways of holding multiple explanations in mind simultaneously. Examples include Marr’s three levels in cognitive science (Marr, 1982), Tinbergen’s four questions in behavioural biology (Tinbergen, 1963), and Giddens’ structuration theory in sociology (Giddens, 1984). To our knowledge, the extent to which that training succeeds has not been studied. However, there is some related research in the domain of psychological disorders. Sufferers from depression become pessimistic about their prognosis when their problems are framed as ‘biological’ (because biological implies non-malleable). Lebowitz et al. (2013) employed a brief audio-visual intervention explaining how brains respond dynamically to environmental inputs. Seeing this intervention reduced prognostic pessimism amongst depression sufferers who endorsed biogenetic explanations, presumably because it broke the intuitive link between ‘biology’ and ‘non-malleable’. It would be interesting to study whether such interventions have a more general effect on people’s perceptions of compatibility, and implied malleability, when biological explanations are given. If successful, such work could suggest pathways to facilitating the acceptance of explanatory pluralism.

## ACKNOWLEDGMENTS

The authors thank Emma Bridger and Eva Wittenberg for their feedback, and Coralie Chevallier and the team Evolution et cognition sociale at the Institut Jean Nicod for their support.

## AUTHOR CONTRIBUTIONS

Contributed to conception and design: DN, WEF, KP. Led the acquisition and analysis of data: DN. Contributed to interpretation of data: DN, WEF, KP. Drafted and/or revised the article, and approved the submitted version for publication: DN, WEF, KP.

## FUNDING INFORMATION

DN’s research is supported by the EUR FrontCog grants ANR-17-EURE-0017 and ANR-10-IDEX-0001-02 to Université PSL; and ANR grant ANR-21-CE28-0009. WEF’s contributions have been supported by the Dutch Research Council (V1.Vidi.195.130) and the James S. McDonnell Foundation (https://doi.org/10.37717/220020502).

## DATA AVAILABILITY STATEMENT

All data and code are available at: https://osf.io/wte2c/.
